# Heroin Relapse "Strikes a Nerve": A Rare Case of Drug-Induced Acute Myelopathy

**DOI:** 10.7759/cureus.15865

**Published:** 2021-06-23

**Authors:** Mandeep K Sidhu, Armugam P Mekala, Joshua A Ronen, Ahmad Hamdan, Sai S Mungara

**Affiliations:** 1 Internal Medicine, Texas Tech University Health Sciences Center School of Medicine at the Permian Basin, Odessa, USA

**Keywords:** opioids, heroin abuse, intravenous drug use, myelopathy, transverse myelitis, opioid-induced myelopathy, heroin-induced myelopathy, opioid epidemic

## Abstract

Opioid addiction is a major public health problem. Through a commitment to individualized treatment plans meant to help patients meet personal goals, behavioral therapy can encourage abstinence and help prevent relapses that can have debilitating consequences.

This case describes a 31-year-old male with heroin relapse who presented with flaccid quadriparesis as well as loss of sensation below the T2-3 spinal level, loss of rectal tone, and urinary retention. A urine drug screen (UDS) was positive for opiates and amphetamines. Autoimmune serologies were negative. Cerebrospinal fluid (CSF) analysis was negative for any acute ongoing infectious process. Magnetic resonance imaging (MRIs) of the cervical and thoracic spine showed increased intramedullary signals with spinal cord expansion from C2-T2, indicating acute transverse myelitis. Upon completion of the aforementioned work-up, idiopathic transverse myelopathy (TM) was diagnosed, and the patient was started on intravenous (IV) methylprednisolone; he also received five sessions of plasmapheresis. By process of elimination, suspicion remained of a diagnosis of opioid-induced myelopathy. The patient showed mild improvement in his original sensory deficits and flaccid quadriplegia.

## Introduction

Transverse myelitis (TM) is described as an inflammation of the spinal cord which can disrupt normal physiologic impulse conduction due to myelin and axonal damage. Gold standard diagnostic criteria include paralysis and sensory loss at multiple levels as well as rapid-onset weakness and bowel or bladder dysfunction in patients without radiographic evidence of a compressive spinal cord lesion [1]. It is considered to be a rare disorder. Generally speaking, most cases are post-infectious secondary to Enteroviruses, West Nile Virus, Herpes Virus, Human Immunodeficiency Virus (HIV), Human T-cell Leukemia Virus type 1 (HTLV-1), *Treponema pallidum* (Syphilis), Lyme disease (more specifically, Neuroborreliosis), and Tuberculosis. Additionally, autoimmune disorders commonly associated with TM include Multiple Sclerosis (MS), Sjogren's syndrome, Systemic Lupus Erythematosus, and Sarcoidosis [1]. Cases of paraneoplastic myelopathy have also been described as secondary to small cell lung cancer (SCLC). However, idiopathic TM accounts for up to 30% of cases. Notwithstanding, heroin-induced myelopathy can also occur in affected patients with prior history of opioid habituation who have been recently re-exposed to heroin after a variable period of abstinence from the drug and could be attributed to hypersensitivity reactions due to prior sensitization [2-3]. While the exact mechanisms are unknown, they could include direct toxicity from heroin - a severe systemic reaction causing temporary vascular insufficiency, especially to the vulnerable circulation in the thoracic cord or arterial embolism [3-5]. Heroin-induced myelitis is typically associated with an acute onset, whereas infectious myelitis tends to have more of a subacute onset [3]. The following describes a case of acute transverse myelitis observed as a complication of intravenous heroin relapse in a young adult.

## Case presentation

A 31-year-old male with a history of intravenous polysubstance abuse (amphetamines, opioids) with recent relapse, presented to an outside facility with flaccid quadriplegia and loss of sensation at and below the level of the nipple line a few hours prior to arrival. He was given one dose of naloxone and transferred to the receiving hospital for a higher level of care. He denied any fever, headaches, shortness of breath, chest pain, aphasia, blurring of vision, recent travel, exposure to toxins, or similar episodes in the past. Initial vitals on presentation were within normal limits except for sinus tachycardia of 126 beats per minute. Cranial Nerves II-XII were intact, bilateral, and symmetric. The patient had notable bilateral upper extremity weakness with +3/5 strength as per the Oxford scale and complete bilateral lower extremity flaccid paralysis. Reflexes were 1+ in the upper extremities and absent in the lower extremities as per the National Institute of Neurological Disorders and Stroke (NINDS) scale. Additionally, there was a complete absence of rectal tone and sensation below the T2-T3 spinal level. Bilateral extensor plantar responses were elicited. Executive functions such as orientation, speech, and comprehension were intact. Pertinent lab findings are shown in Table 1.

**Table 1:** Summary of Laboratory Findings If the displayed values are not otherwise specified as high (H) or low (L) as compared to standard reference laboratory values, they are within normal limits. MRSA (Methicillin-resistant Staphylococcus aureus), PCR (Polymerase Chain Reaction), WBC (White blood cell), RBC (Red blood cell), IgG (Immunoglobulin G)

Serum & Body Fluid Analyses	Results
White Blood (cells/L)	16.4 (H)
Hemoglobin (g/dL)	13.3
Creatinine (mg/dL)	0.7
Aspartate Aminotransferase (U/L)	146 (H)
Alanine Aminotransferase (U/L)	66 (H)
Alkaline Phosphatase (U/L)	87
Bilirubin (mg/dL)	0.4
Creatine Kinase (U/L)	8272 (H)
Thyroid Stimulating Hormone (μU/mL)	0.298 (L)
Free T4 (ng/dL)	0.67 (L)
Acute Hepatitis Panel	Negative
Urine Drug Screen	Positive for amphetamines, benzodiazepines, methamphetamines, and opioids
C-Reactive Protein (mg/dL)	16.6 (H)
Rheumatoid Factor	Negative
Cerebrospinal Fluid (CSF) Analysis	WBC 0-1/μL, RBC 0-10/μL, Segmented WBCs 100/μL (H), Glucose 56 mg/dL, Protein 161.7 mg/dL (H), Myelin basic protein 159.90 (H)
Repeated CSF Analysis (5 days after first)	WBC 6-8/μL, RBC 5-75/μL (H), Segmented WBCs 5-6/μL, Glucose 93 mg/dL (H), Protein 41.6 mg/dL, West Nile IgG 1.38 (H)
MRSA PCR of the nares	Positive

**Figure 1:**
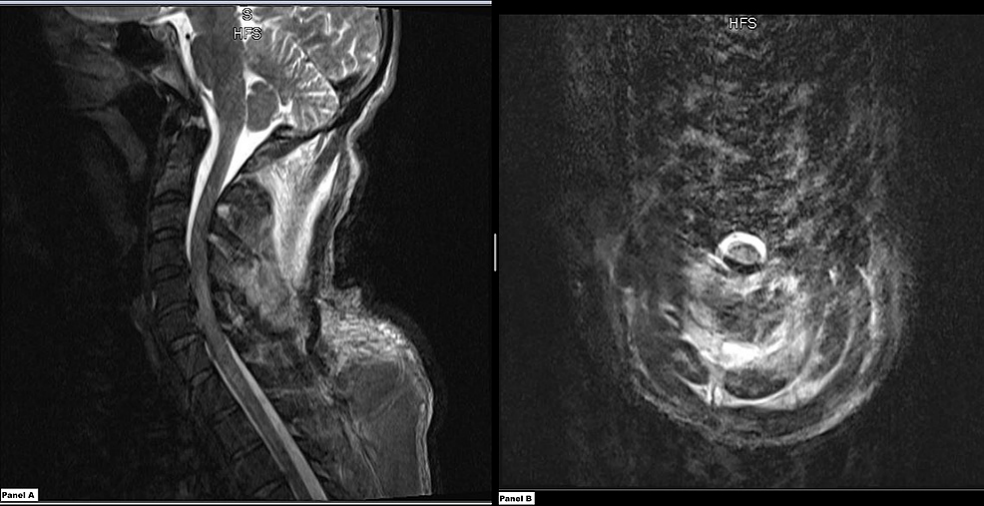
Magnetic Resonance imaging results of the cervical spine Panel A (left): Sagittal section Panel B (right): Axial section at C3-C4 level Impression: There is a diffuse abnormal increased signal on T2-weighted imaging and swelling of the spinal cord extending from C2-T2. These findings are worrisome for acute transverse myelitis. Diffuse hyperemia is seen within the paraspinal musculature on T2-weighted imaging and intermediate signal intensity on precontrast T1-weighted imaging extending from the skull base to C7 bilaterally and within the left serratus anterior muscle.

The Cerebrospinal fluid (CSF) analysis was negative for ongoing infectious processes including West Nile Virus, Haemophilus influenzae, *Streptococcus pneumoniae*, all serotypes of Neisseria Meningitidis, *Escherichia coli*, Group B *Streptococcus*, Epstein Barr Virus, Herpes Simplex Virus, Varicella-Zoster Virus, *Borrelia burgdorferi*, Enterovirus, Cryptococcus, and Coccidiodes. An autoimmune work-up and Human Immunodeficiency Virus testing were also negative. The MRI of the cervical and thoracic spine shown in Figure 1 displayed increased intramedullary signals in T2-weighted images with spinal cord expansion extending from C2 to T2 suggesting transverse myelitis in addition to diffuse hyperemia in paraspinal musculature. In light of this evidence, secondary transverse myelitis in the setting of the patient's recent opioid relapse was suspected and the patient was started on intravenous (IV) methylprednisolone. He also received five sessions of plasmapheresis during the admission at the discretion of Neurology. His hospital course was complicated by methicillin-resistant coagulase-negative *Staphylococcus epidermidis* bacteremia with tricuspid valve endocarditis. He received broad-spectrum IV antibiotics (Vancomycin, Piperacillin-Tazobactam), antifungals (Fluconazole), and anti-virals (Acyclovir) during his hospital stay. He showed mild improvement in his original sensory deficits and upper extremity motor strength. A follow-up spinal cord MRI one week after presentation revealed mild lesion resolution with improvement in edema. The repeat blood cultures for the “test of cure” of his bacteremia were negative. At the time of discharge, he still had unchanged lower extremity flaccid paralysis with urinary retention and loss of rectal tone. He was discharged on six weeks of IV Daptomycin therapy, tapering doses of steroids, a suprapubic catheter, outpatient neurology follow-up instructions, and physiotherapy.

## Discussion

The opioid epidemic is a well-known major public health hazard in the United States which makes it essential to understand the life-threatening manifestations of abuse less commonly highlighted, including heroin-induced myelopathy [6]. Heroin itself is mostly administered intravenously, but other modes of systemic delivery include subcutaneous, intramuscular, or intranasal routes, as Sahni, Garg, Agarwal, and Singh outline [4]. Furthermore, Sporer explains that it acts as a pro-drug that allows rapid central nervous system (CNS) absorption and thus accounts for the drug's euphoric and toxic effects [7]. Heroin use can cause various neurological and non-neurological complications like rhabdomyolysis, compartment syndrome, endocarditis, non-cardiogenic pulmonary edema, acute transverse myelitis, seizures, cerebrovascular accidents, and peripheral nerve lesions [2-4]. While the exact mechanisms are unknown, they could include direct toxicity from heroin - a severe systemic reaction causing temporary vascular insufficiency, especially to the vulnerable circulation in the thoracic cord or arterial embolism. Laboratory signs of rhabdomyolysis were evident in our patient, and routine supportive care with IV fluid resuscitation ensued. Heroin-induced myelopathy is an uncommon complication of heroin use. It is usually reported in individuals recently re-exposed to heroin after a variable drug-free period, indicating an immunopathological cause that is presently not well understood. This indicates some sort of sensitization beforehand, which then results in a hypersensitivity reaction on re-exposure to the drug [3,8]. Spinal cord enhancement on MR imaging, as in this case, suggests inflammation.

High-dose IV steroids are the treatment of choice in acute idiopathic TM as well as acute and recurrent secondary TM [3]. Clinicians do not need to wait for a full workup, including labs and imaging, to be completed prior to initiating therapy. Preferred steroid regimens include methylprednisolone (30 mg/kg up to 1 g daily) or dexamethasone (120 - 200 mg/day for adults) for three to five days [9]. Continuation of therapy depends on clinical course and evident changes in interval radiography. Plasma exchange has also been described in the present literature to be a potentially early adjunct during steroid therapy in acute TM patients with significant deficits, including primary motor impairment, or as a second-line option in steroid-refractory disease as Krishnan and Greenberg summarize [9]. The preferred plasma exchange regimen is five treatments with an exchange of 1.1-1.5 plasma volumes every other day for a total of ten days. While data is lacking on the treatment of opioid-induced myelopathy per se, such treatment ensued in this case per consultant recommendations given the available evidence on IV steroids and plasmapheresis in acute idiopathic and acute and recurrent secondary TM. In recurrent idiopathic TM, immunomodulating therapy with disease-modifying agents such as mycophenolate mofetil or IV rituximab is also a reasonable option [9]. Most notably, patients with acute idiopathic TM and underlying Systemic Lupus Erythematosus (SLE) have been noted to have a great response to one single pulse dose of IV cyclophosphamide. Despite early diagnosis and prompt treatment, heroin-induced myelopathy can lead to acute and complete spinal cord injury with poor longer-term outcomes, as Ivanovski, Espino Ibañez, Barcelo, and Gomila Muñiz suggest [8]. Such patients with poor longer-term outcomes typically experience complete paraplegia and spinal shock. Myelin basic protein (MBP) detected in CSF by enzyme-linked immunosorbent assay (ELISA) testing can help detect central active demyelination such as in multiple sclerosis (MS) and spinal cord demyelination such as in Type I-associated myelopathy/tropical spastic paraparesis (in the setting of HTLV-1 infection) [10]. Only a fraction of patients with TM develop MS or other such neurologic diseases, and MBP has arisen as a marker of acute relapse that rapidly increases, then declines and disappears as Ohta and Ohta elaborate [10]. Definitive diagnosis requires adequate follow-up. Those patients with idiopathic TM may see partial recovery beginning between one to three months after disease onset with adequate therapy and physical rehabilitation. However, the recovery process can still take many years, and persisting disability occurs in close to half of affected patients. 

Heroin addiction is a chronic disease that affects every aspect of a person's life, including job performance, family and social relationships, as well as mental and physical well-being [9]. The social and therapeutic consequences of heroin addiction should always be taken into account per Richter, Pearson, Bruun, Challenor, Brust, and Baden [2]. Medical therapy should be coupled with adequate multidisciplinary rehabilitation and restoration, including supporting patients to build the quality of life they want: physical health, mental and emotional health, and social and occupational functioning [2]. The process of addiction recovery is multifaceted and expands far beyond abstinence versus relapse [8]. Therefore, support must be provided for the establishment of community-based and community-oriented programs for prevention, education, and treatment [2].

## Conclusions

As the opioid epidemic continues, understanding manifestations of abuse including heroin-associated myelopathy remain essential. While respiratory depression remains an ever-present immediate threat to life, heroin-induced myelopathy is an uncommon but often devastating complication of heroin use as well. Additional research is needed to define clinical biomarkers of disease that can help predict outcomes and risk of recurrence. Whether they would be diagnostically beneficial in opioid-induced disease requires further investigation. Acute TM is usually reported in individuals exposed to intravenous heroin after a variable drug-free period, leading to acute and complete spinal cord injury with poor long-term outcomes. Infectious and autoimmune causes should also be considered in differential diagnoses for suspected secondary disease - moreso in the presence of positive bactigen and auto-antibody testing. Nonetheless, early diagnosis with MR imaging and rapid initiation of treatment with high-dose steroids (and adjunctive plasma exchange if needed) may improve outcomes. Complete recovery is rarely reported.
